# Angiography‐guided mid/high septal implantation of ventricular leads in patients with congenital heart disease

**DOI:** 10.1002/joa3.12636

**Published:** 2021-09-17

**Authors:** Jayaprakash Shenthar, Sanjai P. Valappil, Maneesh K. Rai, Bharatraj Banavalikar, Deepak Padmanabhan, Tammo Delhaas

**Affiliations:** ^1^ Electrophysiology Unit Department of Cardiology Sri Jayadeva Institute of Cardiovascular Sciences and Research Bangalore India; ^2^ Department of Biomedical Engineering Maastricht UMC+ Maastricht The Netherlands

**Keywords:** angiography, congenital heart disease, imaging, permanent pacemaker implantation, septal pacing

## Abstract

**Background:**

Conduction system pacing prevents pacing‐induced cardiomyopathy, but it can be challenging to perform in patients with congenital heart disease (CHD), and mid/high septal lead implantation is an alternative. This study aimed to assess intraprocedural angiography's utility as a guide for mid/high‐septal lead implantation in CHD patients.

**Methods:**

The study subjects were CHD patients with Class I/IIa indications for permanent pacemaker implantation. To guide septal lead implantation, we performed an intraprocedural right ventricular angiogram in anteroposterior, 40° left anterior oblique, and 30° right anterior oblique. The primary endpoint was the lead tip in the mid/high septum on computed tomography (CT). The secondary endpoints were complications and systemic ventricular function on follow‐up.

**Results:**

From January 2008 to December 2018, we enrolled 27 patients (mean age: 30 ± 20 years; M:F 17:10) with CHD (unoperated: 20, operated: 7). The mean paced QRS duration was 131.7 ± 5.8 ms, and CT done in 22/27 patients confirmed the lead tip in the mid‐septum in 16, high septum in 5, and apical septum in 1 patient. There were no procedural complications, and during a mean follow‐up of 58 ± 35.2 months, there was no significant change in the systemic ventricular ejection fraction (56.4 ± 8.3% vs 53.9 + 5.9%, *P* = .08). Two patients with Eisenmenger syndrome died because of refractory heart failure.

**Conclusions:**

Intraprocedural angiography is safe and useful to guide mid/high‐septal lead implantation in CHD patients. Mid/high septal lead position preserves systemic ventricular function in patients with CHD during medium‐term follow‐up.

## Graphical abstract

Mid/high septal pacing can be an alternative to right ventricular apical pacing to avoid pacing induced cardiomyopathy. Intraprocedural angiography during permanent pacemaker implantation can define the altered septal anatomy in real time to guide mid/high septal lead placement. Mid/high septal lead placement preserves systemic ventricular function in medium term and is safe mid/high septal lead placement. Mid/high septal lead placement preserves systemic ventricular function in medium‐term and is safe.

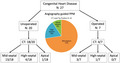

## 1 INTRODUCTION

Atrioventricular block (AVB) and sick sinus syndrome (SSS) can occur in uncorrected congenital heart disease (CHD) or after surgical correction of certain CHDs.^1^ CHD patients requiring pacing therapy often have epicardial leads implanted. Unfortunately, steroid‐eluting epicardial pacing leads tend to fail more often and earlier compared to transvenous pacing leads.^2^ Transvenous pacing is increasingly preferred in CHD patients because it does not need thoracotomy. It is possible to deploy the active fixation at any desired location with good long‐term stability and performance. However, patients with CHD often exhibit altered atrioventricular connections, complex relationships of the cardiac chambers, and distortions of the chamber and vascular anatomy because of surgical procedures, all of which present difficulties and technical challenges for transvenous pacemaker implantation.^3^

Studies have shown that right‐ventricular epicardial and apical endocardial pacing of the venous ventricle causes alteration in myofibrillar architecture, pathologic remodeling, systemic ventricular dilatation, and ventricular dysfunction.^4^, ^5^, ^6^, ^7^ Hence, alternative site pacing is increasingly considered an alternative to transvenous apical pacing. Studies have demonstrated that conduction system pacing preserves systemic ventricular function in patients with structurally normal hearts. Because most CHD patients have gross anatomical abnormalities and variations in the position and course of the conduction system and because of lack of specific implant tools. It may be challenging if not impossible to perform conduction system pacing in patients with CHD.^8^ Hence, in patients with CHD, the preferred lead position has shifted to the high‐ or mid‐septal area of the sub‐pulmonary ventricle.^2^, ^9^

Fluoroscopy, traditionally used for transvenous lead placement, cannot identify the cardiac chambers' complicated anatomical relationship in patients with CHD. Thus, developing a procedure that can delineate the anatomy of the ventricular septum during permanent pacemaker implantation (PPI) is crucial. The present study describes the utility of intraprocedural angiography to guide mid/high‐septal lead implantation as validated by computed tomography (CT) during transvenous PPI in patients with CHD.

## 2 METHODS

### 2.1 Patient selection

We prospectively enrolled patients with uncorrected or surgically corrected CHD with symptomatic bradyarrhythmia and Class I/IIa indication for transvenous permanent pacing.^10^ All procedures were carried out at the Sri Jayadeva Institute of Cardiovascular Sciences and Research, Bengaluru, India. The institutional ethics committee approved the study. Detailed written informed consent was obtained from all patients or from the parents if patients were <18 years of age. We explained the rationale for the procedure, the risks involved, and the novelty of the approach in the patient's local language before enrollment. Exclusion criteria were systemic/arterial ventricle ejection fraction (EF) of <40%, contrast allergy, pregnancy, and renal dysfunction.

We collected a thorough clinical history from each participant, including details of previous surgical procedures (if any), physical examination, blood chemistry, renal function tests, 12‐lead electrocardiogram, and 24‐hour Holter or extended external loop recording if necessary. An experienced pediatric cardiologist performed a transthoracic echocardiogram to describe the underlying anatomy, relationship of the various chambers and the great vessels, presence or absence of intracardiac shunts, and systemic ventricular function. A pre‐procedural review of the surgical notes was carried out in patients with previous surgical intracardiac repair to correct CHD.

#### 2.1.1 Endpoints

The primary endpoint was successful implantation of the ventricular lead in the mid/high septal position with no periprocedural complications. The secondary endpoint was preserved systemic ventricular function over medium‐term follow‐up, absence of complications such as heart failure, lead‐related issues, and death related to the procedure.

### 2.2 Implantation technique

Implantation procedures were carried out either under conscious sedation, or general anesthesia, after 6 hours of fasting and intravenous hydration with normal saline at 1 mL/kg/min. Venous access was obtained through a single or two separate punctures of the extrathoracic axillary vein for single‐ or dual‐chamber pacemaker implantation. Angiography was carried out using the femoral approach for a single‐chamber pacemaker or through the access for the atrial lead for dual‐chamber pacemaker implantation. A 7‐F peel away introducer (PLI) was used to implant the pacing leads, and a 6/5‐F angiographic sheath (AVANTIR+; Cordis Corporation) with a sidearm was used to introduce an angled 6/5‐F pigtail catheter for angiography. An active‐fixation pacing lead (Medtronic 4076; Medtronic Inc.) along with a stylet was introduced into the right heart through the PLI along with a 6/5‐F pigtail/balloon floatation angiographic catheter positioned in the right atrium or right ventricle depending on the anatomy. Angiography was performed using 30‐40 mL (or 1 mL/kg) of non‐ionic contrast agent (Iohexol) using a flow injector at a flow rate of 12‐15 mL/s and a maximum pressure of 1000 psi. Films were acquired at 15 frames/s in the anteroposterior, 40° left anterior oblique (LAO), and 30° right anterior oblique (RAO) views to define the position and morphology of the sub‐pulmonary ventricle and to define the interventricular septum. The acquired angiograms were used as a guide to target the ventricular lead to the mid/high septum of the sub‐pulmonary ventricle using a stylet, which was manually shaped depending on the anatomy. The definition of the outflow tract, high‐, mid‐, and low septum was done according to the description by Lieberman et al.^11^ The lead was positioned in the apical septum in case of difficulty in achieving a stable mid/high septal lead position because of ventricular septal defect patch repair or other anatomical reasons. The lead tip position was confirmed using fluoroscopy, and if necessary, we performed a repeat angiogram in the view that best defined the septum. Cine runs were performed at 15 frames/s; the rest was done using fluoroscopy at 3.5 frames/s to minimize radiation exposure. Immediately after pacemaker implantation, a 12‐lead electrocardiogram was performed on an electrophysiology recorder (EP tracer; Schwarzer Cardiotek GmbH) after programming the device to single chamber ventricular pacemaker (VVI) at the lowest possible rate to pace the ventricle with no fusion beats. The QRS duration was measured using electronic calipers at a sweep speed of 100 mm/s. After regular post‐procedural care, CT images were acquired to validate the ventricular lead position using a Philips 64‐slice CT scanner (Koninklijke Philips N.V.). A radiologist who was blinded to the procedure validated the position of the ventricular lead tip. The accuracy of the ventricular lead tip position during angiography was compared with the CT images.

### 2.3 Statistical analysis

Continuous variables are expressed as mean ± SD, and categorical data are expressed as numbers or percentages. Continuous data were analyzed using the paired *t* test while comparing the parameters within the same group and unpaired Student's *t* test for comparing the parameters between the groups. Fisher's exact test was used to evaluate dichotomous variables. A *P* < .05 was considered statistically significant. All statistical analyses were performed using SPSS software version 22 (SPSS, Inc.).

## 3 RESULTS

### 3.1 Study population

**CHART 1:**
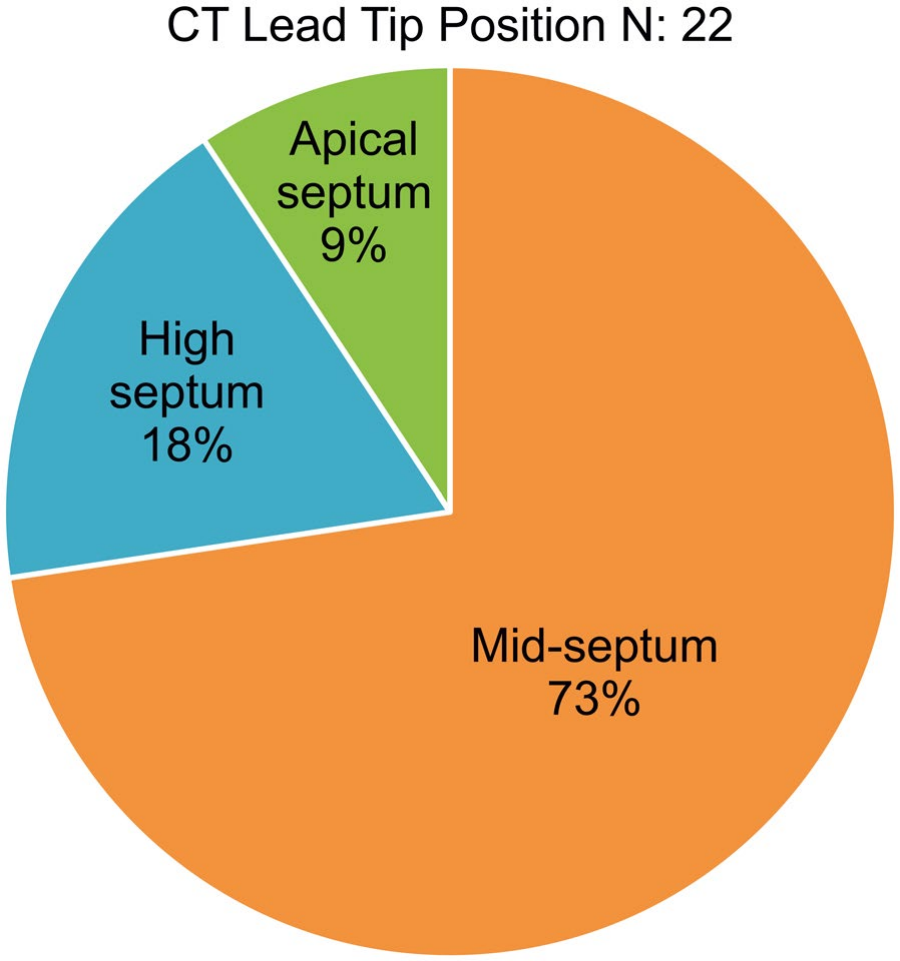
Pie diagram showing lead tip position. The chart shows the final lead tip position in 22 of the 27 patients who underwent computed tomography. The majority (91%) of the leads were placed in the mid/high septum. The lead tip was positioned in the apical septum in 9% for anatomical reasons.

**FIGURE 1:**
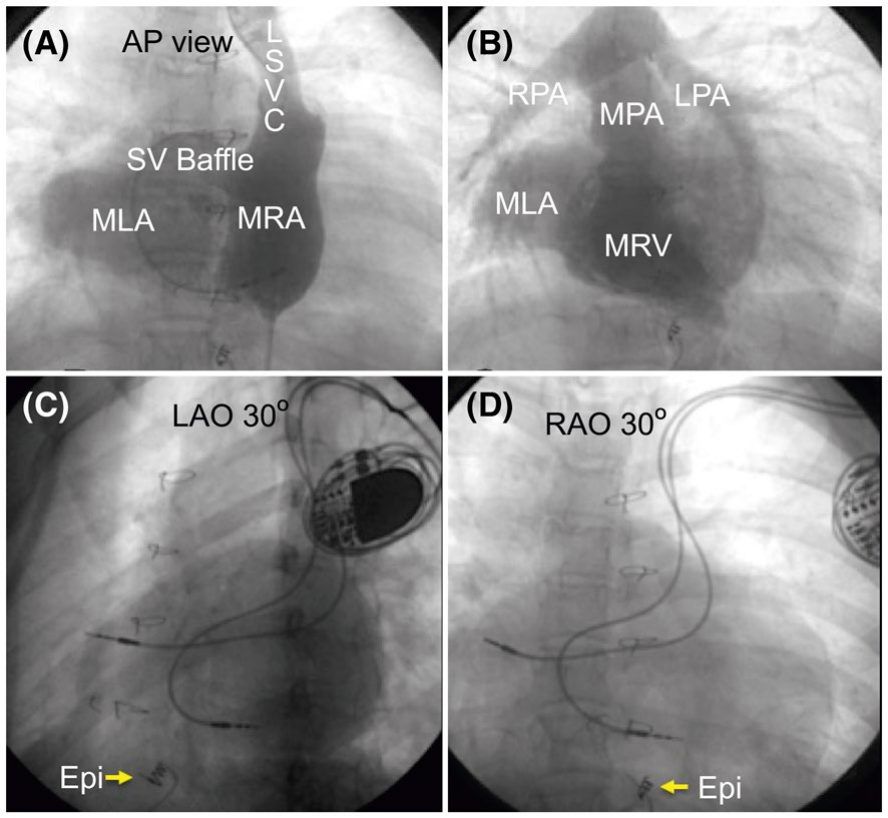
Angiogram of dual‐chamber pacemaker situs inversus, d‐transposition of great arteries, double‐outlet right ventricle, and cardiac total anomalous pulmonary venous connection. 13‐year‐old female patient post arterial switch, atrial septectomy, Senning procedure with ventricular septal defect closure and malfunctioning epicardial VVI pacemaker. (A) shows the angiogram's AP view depicting the MRA, systemic venous baffle to the MLA. (B) illustrates the angiogram in RAO 30° the morphologic left atrium, morphologic right ventricle right pulmonary arteries. (C, D) Fluoroscopy in the LAO and RAO views, showing the final ventricular lead tip position in the mid‐septal area of the morphologic right ventricle. The yellow arrow (Epi) shows the position of the epicardial lead. AP, anteroposterior; AV, atrioventricular; Epi, epicardial pacing lead; IVC, inferior vena cava; LAO, left anterior oblique; LPA, left pulmonary artery; LSVC, left superior vena cava; MLA, morphologic left atrium; MPA, main pulmonary artery; MRA, morphologic right atrium; MRV, morphologic right ventricle; RAO, right anterior oblique; RPA, right pulmonary artery; SV, systemic venous

**FIGURE 2:**
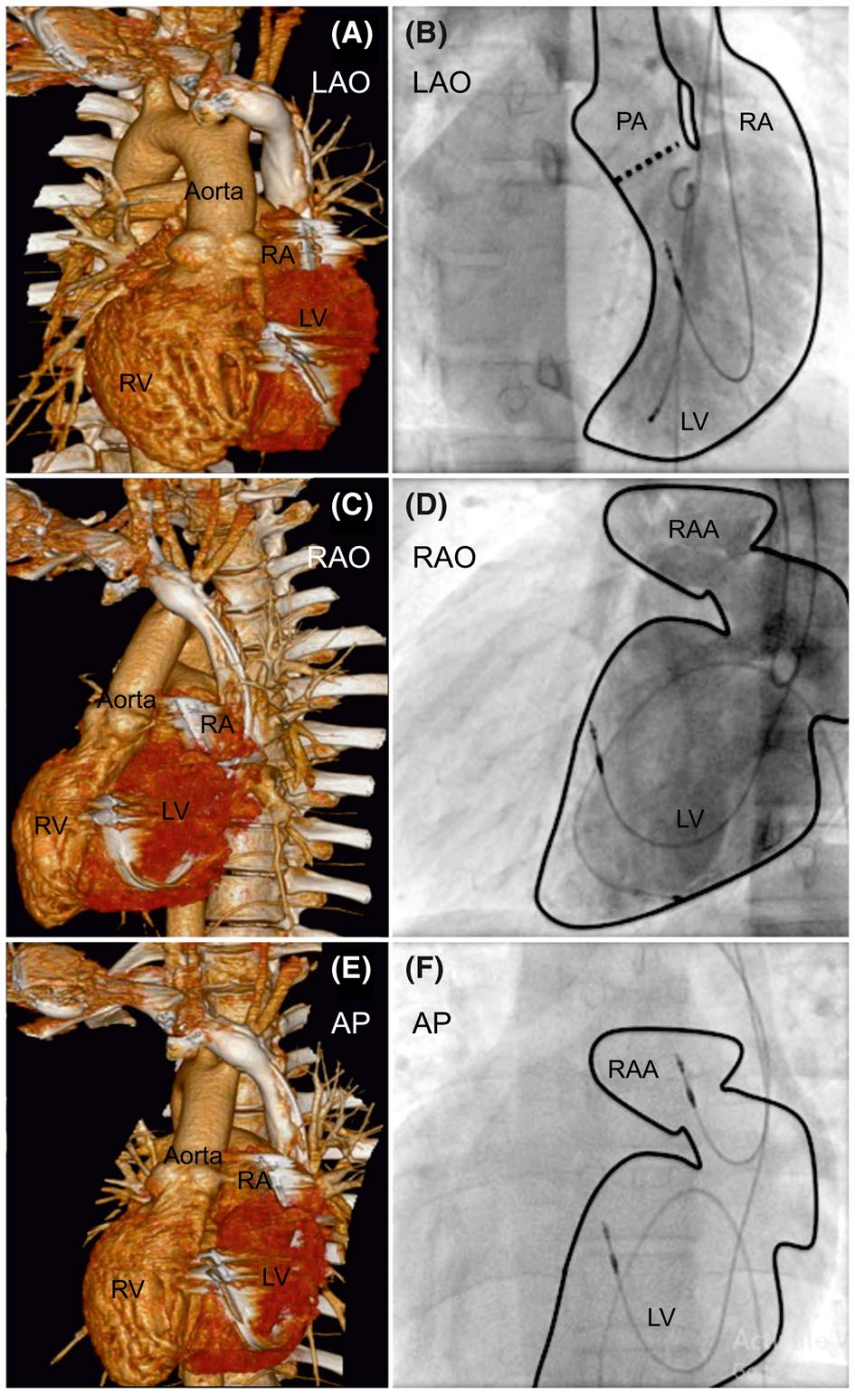
Computed tomographic reconstruction and angiogram of the dual‐chamber pacing system in a patient with situs inversus, dextrocardia, and corrected transposition of the great arteries. The left panels show computed tomographic three‐dimensional reconstructions of the heart. (A) Left anterior oblique, (C) Right anterior oblique, and (E) Anteroposterior views with the pacing lead tip position. Right panels (B), (D), and (F) show procedural angiographic images in the same views as (A), (C), and (E), showing ventricular lead position in the mid‐septal area in the morphological left ventricle, as confirmed by cardiac computed tomography. AP, anteroposterior; LAO, left anterior oblique; LV, left ventricle; RA, right atrium; RV, right ventricle; RAA, right atrial appendage; RAO, right anterior oblique; PA, pulmonary artery

**FIGURE 3:**
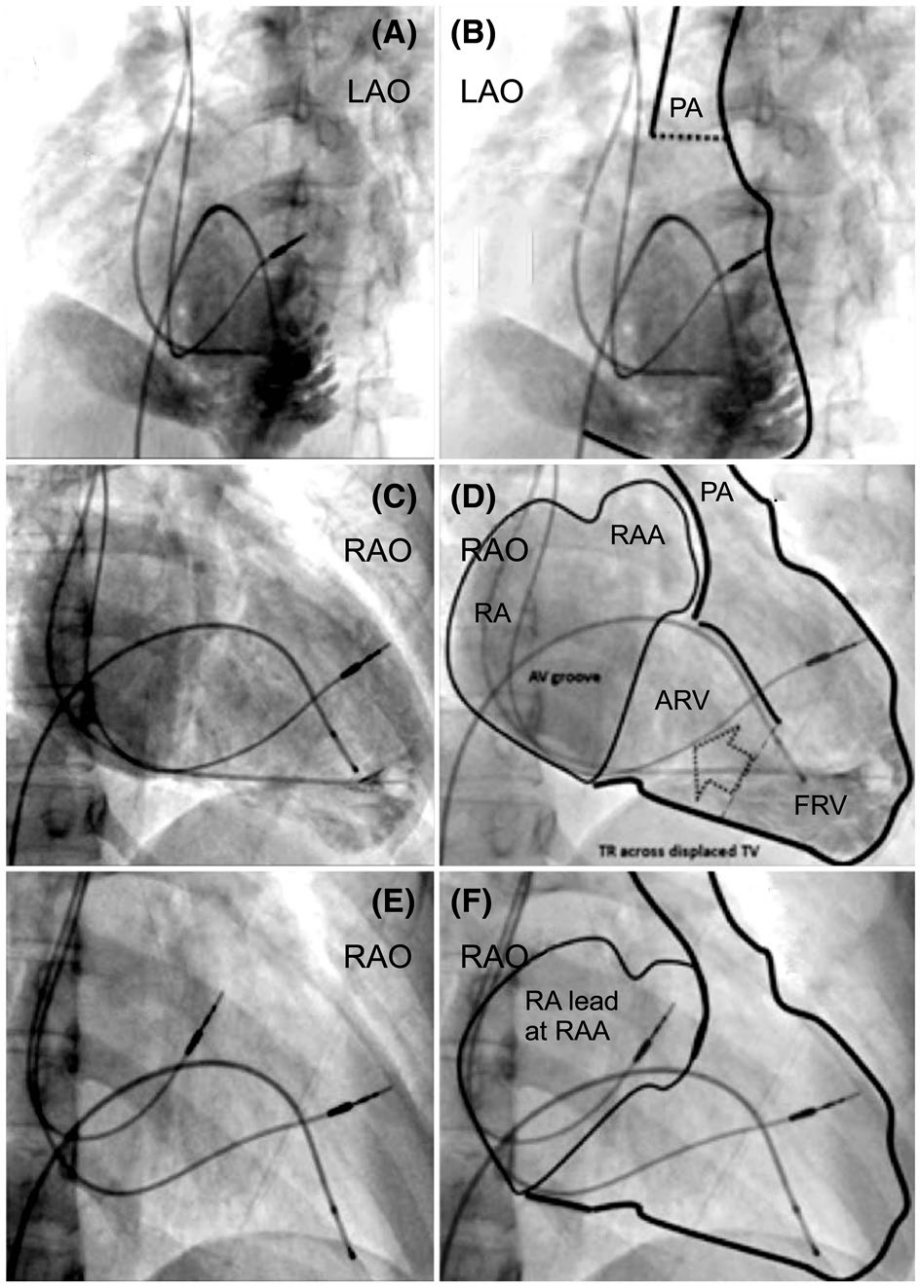
Angiogram of the dual‐chamber pacing system in a patient with Ebstein's anomaly. (A) LAO and (C) RAO view angiograms during ventricular lead placement. (E) The final lead position in the right anterior oblique view. (B), (D), and (F) Angiograms with outlines of the chambers illustrated. The ventricular lead is beyond the displaced tricuspid valve in the high septal position. ARV, atrialized right ventricle; FRV, functional right ventricle; LAO, left anterior oblique; PA, pulmonary artery; RA, right atrium; RAA, right atrial appendage; RAO, right anterior oblique; RV, right ventricle

**FIGURE 4:**
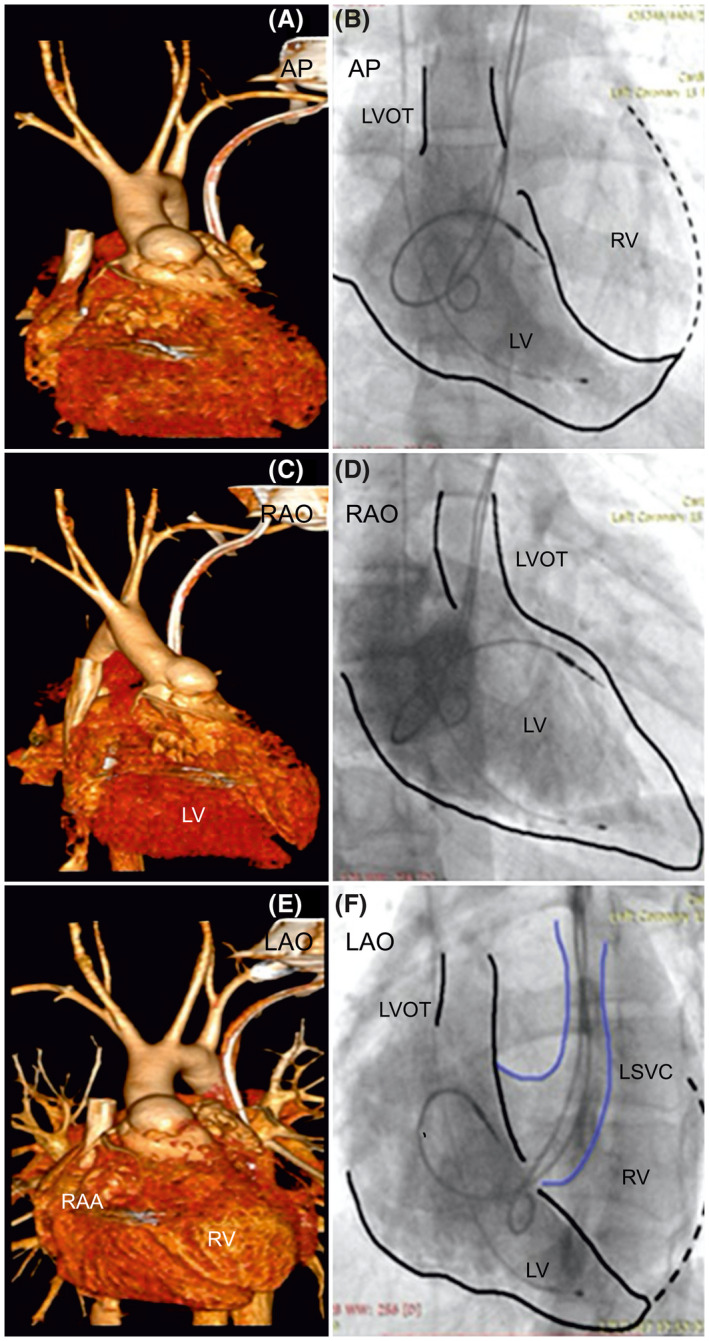
Three‐dimensional reconstruction of computed tomography and angiogram of the dual‐chamber pacing system in a patient with congenitally corrected transposition of the great arteries with persistent left superior vena cava. Computed tomographic three‐dimensional reconstructions of the heart in (A) AP, (C) RAO, and (E) LAO views showing pacing lead tip position. (B), (D), and (F) show procedural angiograms in equivalent views as the top panel. The ventricular lead is in the high‐septal location in the morphological left ventricle, and the atrial lead is in the right atrial appendage. AP, anteroposterior; LAO, left anterior oblique; LV, left ventricle; LVOT, left ventricular outflow tract; LSVC, left superior vena cava; RAA, right atrial appendage; RAO, right anterior oblique; RV, right ventricle

**FIGURE 5:**
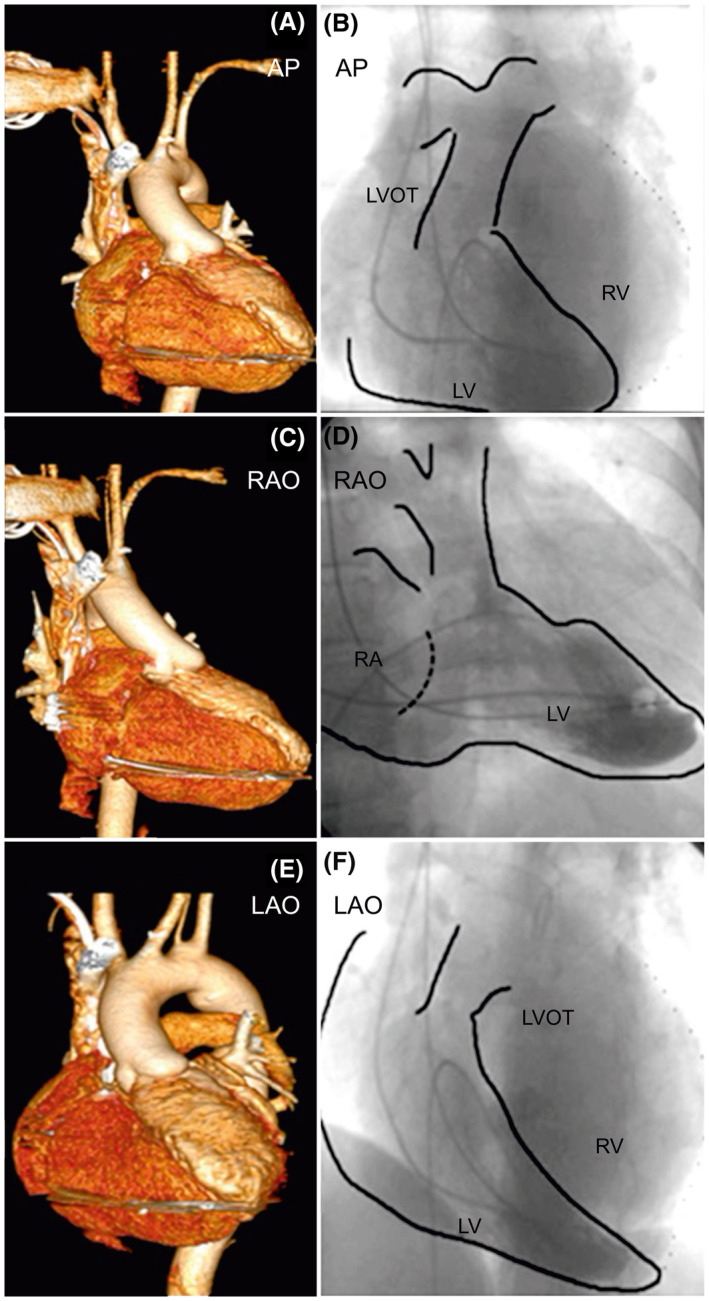
Three‐dimensional reconstruction of computed tomography and angiogram of the dual‐chamber pacemaker in a patient with congenitally corrected transposition of the great arteries, inlet ventricular septal defect, and Eisenmenger syndrome. Computed tomographic three‐dimensional reconstructions of the heart in (A) AP, (C) RAO, and (E) LAO views with the pacing lead tip position. (B), (D), and (F) show procedural angiograms in equivalent views as the left panel, demonstrating ventricular lead position at the apical septal location in the morphological left ventricle. AP, anteroposterior; LAO, left anterior oblique; LV, left ventricle; LVOT, left ventricular outflow tract; RA, right atrium; RAO, right anterior oblique; RV, right ventricle

Between January 2008 and December 2018, we recruited 27 patients with CHD for angiography‐guided lead placement (unoperated: 20, operated: 7). The characteristics of the study population are shown in Table 1 (unoperated) and Table 2 (operated). The total cohort's mean age was 30 ± 20 years, with 63% men (man:woman, 17:10). The mean weight was 48.9 ± 22.1 kg (range 2‐65 kg), and the mean EF of the systemic ventricle was 56.7 ± 7.2% (range: 40%‐65%). The indication for PPI was complete AVB in 24 and SSS in three patients. Eighteen patients were implanted with DDD, and nine with VVI pacemaker. Of the 27 patients, 10 patients had situs inversus with dextrocardia, 17 had situs solitus (2 with dextrocardia). Associated cardiac anomalies included congenitally corrected transposition of the great arteries in 18 patients, dextro‐transposition of the great arteries in two, tetralogy of Fallot (TOF) in four, total anomalous pulmonary venous connection (TAPVC) in three, left atrial isomerism in one, double‐outlet right ventricle in one, and double inlet left ventricle (single‐ventricle physiology) in one. Persistence of the left superior vena cava was observed in two patients. There were two patients with Eisenmenger's syndrome: one patient with situs solitus, double‐inlet left ventricle (single‐ventricle physiology), and d‐transposition of great arteries (D‐TGA); the other patient had situs solitus, congenitally corrected transposition of great arteries (CCTGA), and large‐inlet ventricular septal defect. All patients underwent successful pacemaker implantation with the ventricular lead in a stable position with no perioperative lead dislodgements. The mean fluoroscopic time was 11.1 ± 3.5 minutes (range: 8‐24 minutes), the mean amount of contrast used was 93.1 ± 24.1 mL (range: 33‐120 mL), with a mean paced QRS duration of 131.7 ± 5.8 ms. The mean pre‐and post‐procedural serum creatinine levels were 1.0 ± 0.18 and 1.1 ± 0.13 mg/dL respectively (*P* = .02). We performed CT in 22 of the 27 patients. Five patients (two unoperated and three operated) refused CT examination. The lead tip was in the mid‐septum in most patients, as validated by CT images (Chart 1). In the 22/27 patients who underwent post‐procedure CT, the lead tip was in the mid‐septum in 16 (Figures 1 and 2), high septum in 5 (Figures 3 and 4), and apical septum in 1 (Figure 5), all concordant with the angiographic lead position. In two patients with Eisenmenger syndrome, the lead had to be positioned in the apical septum (Figure 5) because the lead position was unstable in other areas: a large inlet ventricular septal defect in one and a single‐ventricle physiology in the other. There were no patients with leads in the anterior wall, free wall, or outflow tract of the sub‐pulmonary ventricle. There were no procedure‐related complications. Interrogation of the device on the last follow‐up revealed an atrial threshold of 1.2 ± 0.4 V, a ventricular threshold of 0.8 ± 0.6 V at 0.4 ms pulse width, with 100% right ventricular (RV) pacing in patients with complete AV block (24/27) and 38% RV pacing in SSS (3/27). The sensed P wave was 2.3 ± 0.7 mV, and patients with complete AV block had no measurable R waves and were completely pacing dependent. The mean follow‐up duration was 58 ± 35.2 months. There were two deaths in patients with Eisenmenger syndrome not related to the procedure. There was no significant change in the EF 56.7 ± 7.2% vs 54.8 ± 4.8% (*P* = .10), heart failure, lead‐related complications, or deaths related to the procedure.

**TABLE 1:** Demographic, anatomical, clinical, and procedural characteristics of 20 unoperated patients

No.	CHD	Age (y)	Gender	Wt (kg)	EF (%)	Indication	PPM device	Dye (mL)	Fluoro (min)	QRS (ms)	F/U (mo)	CT	Lead position (angio)
1	SI/DC/CCTGA	57	M	70	60	AVB	DDD	100	10	132	75	Y	HS
2	SI/DC/CCTGA	39	M	65	58	AVB	DDD	100	9	128	115	Y	MS
3	SI/DC/CCTGA	52	F	62.3	65	AVB	DDD	100	7	142	101	Y	HS
4	SI/DC/CCTGA	49	M	57	50	AVB	DDD	100	11	136	100	Y	MS
5	SI/DC/CCTGA	49	F	74	55	AVB	VVI	100	10	136	98	Y	MS
6	Ebstein's anomaly	60	F	62.3	60	AVB	VVI	120	9	122	92	Y	HS
7	SS/CCTGA	7	M	20.2	60	AVB	VVI	80	7	120	84	Y	MS
8	SS/CCTGA	7	M	20.2	60	AVB	VVI	80	13	138	72	Y	MS
9	SS/DC/CCTGA	62	M	58	65	AVB	DDD	120	10	132	62	Y	MS
10	SS/CCTGA	35	M	65	55	AVB	DDD	80	8	128	55	Y	MS
11	SS/CCTGA	40	M	48.7	66	AVB	DDD	100	9	138	56	Y	HS
12	SS/Ebstein's anomaly	26	F	48.2	58	AVB	DDD	100	15	138	32	Y	MS
13	SS/DILV/D‐TGA/Eisenmenger syndrome	22	M	74	38	AVB	DDD	120	12	142	8	N	AS
14	CCTGA/LSVC	21	M	58	55	AVB	DDD	100	10	132	28	Y	MS
15	SI/DC/CCTGA	31	F	65	42	SSS	DDD	120	10	134	25	Y	MS
16	L‐TGA/Inlet VSD/Eisenmenger syndrome	40	F	70	40	AVB	VVI	120	15	132	16	Y	AS
17	SI/DC/CCTGA	26	M	68.5	55	AVB	DDD	100	11	134	15	Y	MS
18	SS/CCTGA	2	F	8.5	68	AVB	VVI	100	8	132	17	N	MS
19	SI/DC/CCTGA	55	M	68.5	58	AVB	DDD	100	9	136	12	Y	MS
20	SI/DC/CCTGA	65	M	72	60	SSS	DDD	100	11	134	10	Y	MS
	Mean ± SD	37.3 ± 19.2		56.8 ± 19	54.4 ± 8.3			98.8 ± 19.6	10.2 ± 2.2	133.3 ± 5.7	53.5 ± 36.5		

Abbreviations: AS, apical septum; AVB, atrioventricular block; CCTGA, congenitally corrected transposition of the great arteries; CHD, congenital heart disease; CT, computed tomography; DDD, dual chamber pacemaker; DC, dextrocardia; DILV, double inlet left ventricle; L‐TGA, Levo‐transposition of the great arteries; EF, ejection fraction; F, female; F/U, follow‐up; HS, high septum; LC, levocardia; LSCV, left superior vena cava; M, male; MS, mid‐septum; N, no; PPM, permanent pacemaker; SI, situs inversus; VSD, ventricular septal defect; VVI, single chamber ventricular pacemaker; Y, yes.

**TABLE 2:** Demographic, anatomical, clinical, and procedural characteristics of seven operated patients

No.	CHD	Age (y)	Gender	Wt (kgs)	Sx (d)	EF (%)	Indication	PPM device	Dye (mL)	Fluoro (min)	QRS (ms)	F/U (mo)	CT	Lead position (angio)
1	SS/TOF	7	M	18.6	15	58	AVB	DDD	80	9	126	130	Y	MS
2	SS/TOF	2	M	7.2	60	58	AVB	VVI	33	12	122	77	N	MS
3	SS/CCTGA/ TOF	16	F	39.3	15	56	AVB	DDD	80	13	124	74	N	MS
4	SS/TOF	11	M	32	12	58	AVB	VVI	80	12	130	66	Y	MS
5	SS/LA isomerism/TAPVC/interrupted IVC	12	F	38	90	55	SSS	DDD	120	9	126	54	Y	MS
6	SI/DC/D‐TGA /DORV/TAPVC	13	F	41	11 y	56	AVB	DDD	36	24	130	58	N	MS
7	SS/DC/Cardiac TAPVC	4	M	11.2	15	62	AVB	VVI	45	17	132	33	Y	HS
	Mean + SD	9.3 ± 5.1		26.8 ± 14.1	34.5 ± 32.8	57.6 ± 2.3			67.7 ± 31.4	13.7 ± 5.2	127.1 ± 3.6	70.3 ± 30.1		

Abbreviations: AVB, atrioventricular block; CCTGA, congenitally corrected transposition of the great arteries; CHD, congenital heart disease; CT, computed tomography; DC, dextrocardia; DDD, dual chamber pacemaker; DORV, double outlet left ventricle; D‐TGA, dextro‐transposition of the great arteries; EF, ejection fraction; F, female; F/U, follow‐up; HS, high septum; LA, left atrium; LC, levocardia; M, male; MS, mid‐septum; N, no; PPM, permanent pacemaker; SI, situs inversus; SS, situs solitus; SSS, sick sinus syndrome; TAPVC, total anomalous pulmonary venous connection; TOF, tetralogy of Fallot; VSD, ventricular septal defect; VVI, single chamber ventricular pacemaker; Y, yes.

### 3.2 Unoperated patients (n = 20)

Table 1 presents the characteristics of unoperated patients. Indications for PPI were complete AVB in 18 and SSS in two; of these, DDD was implanted in 14 and VVI in six. Mean pre‐ and post‐procedural serum creatinine levels were 0.9 ± 0.3 and 1.0 ± 0.2 mg/dL respectively (*P* = .11). We performed CT examination in 18/20 patients. The lead was in the mid‐septum in 13 (72.2%), high septum in four (22.2%), and apical septum in one (5.6%). The two patients who refused CT scans were a 2‐year‐old post child and a patient with Eisenmenger syndrome. During a mean follow‐up of 53.5 ± 36.5 months, there was no significant change in the EF 56.4 ± 8.3% vs 53.9 ± 5.9% (*P* = .08). Two patients with Eisenmenger syndrome died because of intractable heart failure, one 8 months and another 13 months after the procedure.

### 3.3 Operated patients (n = 7)

The characteristics of operated patients are presented in Table 2. Operated patients were significantly younger than unoperated patients (9.3 ± 5.1 years vs 37.3 ± 19.2 years, respectively, *P* < .001), with lower mean body weight (26.8 ± 14.1 kg vs 56.8 ± 19 kg, respectively, *P* < .001), and narrower mean QRS duration (127.1 ± 3.6 ms vs 133.3 ± 5.7 ms, respectively, *P* = .002). All AVBs were because of surgical trauma to the AV node, which had not recovered 10 or more days after surgery. The mean time to pacemaker implantation following cardiac surgery was 34.5 ± 32.8 days (range: 12‐90 days) in the six patients who underwent this surgery. There was one female patient with situs inversus, d‐transposition of great arteries, double‐outlet right ventricle, and cardiac total anomalous pulmonary venous connection. She had undergone an arterial switch, atrial septectomy, Senning procedure with the ventricular septal defect closure at 1 year of age. She had a post‐operative AV block for which an epicardial VVI pacemaker implantation had been done a then presented with epicardial pacing lead malfunction at the age of 13 years (Figure 1). The indication for PPI was complete AVB in six patients and SSS in one; of these, DDD was implanted in four and VVI in three patients. The mean fluoroscopy time (13.7±5.2 minutes; range 9‐24 minutes) and mean paced QRS duration (127.1 ± 3.6 ms) were comparable in both unoperated and operated patients. The mean amount of contrast used (67.7 ± 31.4 mL; range: 33‐120 mL) was considerably less in operated patients because of the younger age and lower weight. Mean pre‐ and post‐procedural serum creatinine levels were 0.8 ± 0.16 and 0.9 ± 0.2 mg/dL, respectively (*P* = .16). We performed CT examination in 4/7 patients as three patients refused CT scans. The lead tip was in the mid‐septum in three (75%) and high‐septum in one (25%). There was no significant difference in EF (57.6 ± 2.3% vs 55.8 ± 3.1%; *P* = .11) or deaths during a mean duration of follow‐up duration of 70.3 ± 30.1 months.

Operated patients were significantly younger than unoperated patients (9.3 ± 5.1 years vs 37.3 ± 19.2 years, respectively, *P* = .000002), with lower mean body weight (26.8 ± 14.1 kg vs 56.8 ± 19 kg, respectively, *P* = .0003), and narrower mean QRS duration (127.1 ± 3.6 ms vs 133.3 ± 5.7 ms, respectively, *P* = .002). The mean fluoroscopy time of operated and unoperated patients was not significantly different (13.7 ± 5.2 minutes vs 10.2 ± 2.2 minutes, respectively, *P* = .77). The AVBs were a result of surgical trauma to the AV node, which had not recovered 10 or more days after surgery.

## 4 DISCUSSION

The present study is the first to investigate angiography‐guided septal lead implantation validated by CT data in various operated and unoperated CHD. The following are the salient findings of the present study. Transvenous pacing with mid/high‐septal lead implantation can be successfully performed in patients with various CHDs using venous angiography. Angiography is safe, can be performed with minimal additional equipment, and is available for immediate review to guide lead placement. Even though the operated patients were significantly younger with lower body weight, the procedural time was not different. Mid/high septal lead implantation in patients with CHD preserves systemic ventricular function, avoids heart failure, and has no lead‐related complications on medium‐term follow‐up.

There is a high incidence of lead failures in pediatric pacing patients, most common in younger patients, those with CHD, and with epicardial lead systems. Hence, increased utilization of transvenous systems in smaller patients is justified when anatomy permits.^9^ A complete understanding of the cardiovascular anatomy is critical before implanting a permanent transvenous pacemaker in patients with congenital CHD. Implantation can pose considerable difficulties in such patients because of the altered anatomy, exacerbated if the patient has undergone a previous surgical correction.^12^, ^13^ A contractility‐guided study indicated that mid/high‐septal ventricular implant sites might offer the best‐paced ventricular contractility and better ventricular synchrony than the apical septum.^14^, ^15^ However, implanting a lead in the mid‐septal position using fluoroscopic landmarks alone is often inaccurate, even in patients with normal anatomy.^16^, ^17^, ^18^ The reliance on fluoroscopy alone to guide the positioning of the lead in patients with complex CHD makes implantation challenging because fluoroscopy reveals only the cardiac silhouette without details of the complex intracardiac and venous anatomy. Although preoperative echocardiogram, CT, and magnetic resonance imaging can define the cardiac anatomy^19^ they do not provide real‐time imaging during the procedure.

The present study highlights the utility of angiography during PPI in patients with CHD, supporting previous studies on angiography in pacemaker implantation in such patients.^20^, ^21^, ^22^ Angiography‐guided PPI requires minimal additional equipment (except for the pressure injector), is relatively cheap, and requires acceptable fluoroscopy and procedure times. Angiography provides valuable real‐time information on cardiac anatomy and the orientation of the mid‐septal area. It reveals the vascular course, including the conduits or baffles, that needs to be navigated by the lead.

A recent study reported physiological pacing (His bundle or left bundle ) in congenitally corrected transposition of great arteries in 15 patients from 10 centers with a success rate of 86%.^23^ However, selective pacing was only possible in three of 13 patients even after 3D electroanatomical mapping, and the paced QRS showed incomplete or complete left bundle branch block in all patients.^23^ In the multicenter study in CHD patients, prolonged fluoroscopy was noted with a median fluoroscopy time of 38 minutes (range: 19‐62 minutes).^23^ In the present study, the fluoroscopic time was 11.1 ± 3.5 minutes (range: 8‐24 minutes) in comparison.

Although permanent His bundle pacing is ideal, this approach is limited in CHD patients because there is a lack of specific hardware for their altered anatomy. towing to the high pacing thresholds in His bundle pacing, there is also a necessity for early pulse generator replacement, even in patients with normal anatomy.^8^, ^24^ The other drawbacks of 3D‐electroanatomical mapping‐guided PPI include the high cost of the procedure, the requirement of 3D‐mapping equipment, and personnel with expertise to perform both procedures. It may also be challenging to achieve long‐term stability of physiological pacing because of the wide variations in the conduction system anatomy in CHD patients.

As noted in the present study, mid/high septal pacing did not cause systemic ventricular dysfunction, heart failure, or lead‐related complications over medium‐term follow‐up. Considering the highly variable anatomical course of the AV node and His‐Purkinje system, mid/high‐septal pacing of the sub‐pulmonic ventricle is likely to be a better option for CHD patients.

## 5 LIMITATIONS

The present study is a single‐center study involving a group of patients with CHD who underwent PPI. Further studies are needed in a larger number of patients to validate the results of the present study. The current study results do not apply to those patients in whom the venous access is from the femoral, iliac, and hepatic veins. The procedure cannot be performed in patients with contraindications to angiography. The implanting physician should be familiar with the anatomy of CHD.

## 6 CONCLUSIONS

Transvenous mid/high‐septal pacing may be optimal for CHD patients requiring pacing therapy for bradyarrhythmia because of the lead stability, long‐term performance, and patient outcomes associated with the procedure. Intraprocedural angiography is beneficial for such patients as it provides real‐time cardiac and vascular anatomy data to guide mid/high‐septal lead implantation. Angiography is a safe imaging modality for septal lead placement in patients with CHD. Larger multicenter studies are required to confirm the utility of angiography‐guided mid/high‐septal pacing in patients with CHD and to further evaluate patient outcomes.

## CONFLICT OF INTEREST

Authors declare no conflict of interests for this article.

## AUTHOR CONTRIBUTIONS

Jayaprakash Shenthar: Concept/design, data analysis/interpretation, drafting article, critical revision of article, approval of article, statistics, funding secured by, and data collection; Sanjai Pattu Valappil: Data analysis/interpretation, critical revision of the article, approval of article, statistics, and data collection; Maneesh K Rai: Data analysis/interpretation, drafting article, critical revision of the article, approval of article, statistics, and data collection; Bharatraj Banavalikar: Data analysis/interpretation, critical revision of the article, approval of article, statistics, and data collection; Deepak Padmanabhan: Data analysis/interpretation, critical revision of the article, approval of article, statistics, and data collection; Tammo Delhaas: Concept/design, drafting article, critical revision of the article, and approval of article.

## Data Availability

All data are incorporated into the article.
